# Effects of housing systems and laying phases on external and internal egg quality characteristics of indigenous guinea fowl hens

**DOI:** 10.1093/tas/txae011

**Published:** 2024-01-22

**Authors:** Olubukola P A Idowu, Damilola U Kareem, Oyegunle E Oke, Emmanuel A Adeyeye, Olajide M Sogunle, Olusegun M O Idowu

**Affiliations:** Agricultural Media Resources and Extension Centre, Federal University of Agriculture, Abeokuta, P.M.B. 2240, Nigeria; Department of Animal Production and Health, Federal University of Agriculture, Abeokuta, P.M.B. 2240, Nigeria; Department of Animal Nutrition, Federal University of Agriculture, Abeokuta, P.M.B. 2240, Nigeria; Department of Animal Physiology, Federal University of Agriculture, Abeokuta, P.M.B. 2240, Nigeria; Department of Animal Nutrition, Federal University of Agriculture, Abeokuta, P.M.B. 2240, Nigeria; Department of Animal Production and Health, Federal University of Agriculture, Abeokuta, P.M.B. 2240, Nigeria; Department of Animal Nutrition, Federal University of Agriculture, Abeokuta, P.M.B. 2240, Nigeria

**Keywords:** battery cage, deep litter, egg quality, free run, interaction

## Abstract

The study investigated the effect of housing system and laying phases on the internal and external qualities of guinea fowl eggs laid in three different housing systems. The trial involved the use of 117, 34-wk-old guinea fowl hens which were assigned to three housing types, which were battery cages, deep litter, and deep litter housing systems with free runs, which had the same dimensions as deep litter houses but with free run and open-air space to allow the birds to have free access to soil and exhibit natural behavior. The laying phases of the birds were partitioned into three (36 to 40, 41 to 45, and 46 to 50 wk of age). The data collected were subjected to analysis of variance (ANOVA) using a 2 × 3 factorial design. The findings demonstrated that both housing type and laying period significantly influenced egg quality parameters, with deep litter housing exhibiting higher egg weight, egg breadth, and shell surface area. Battery cage housing had thicker shells and a higher percentage of shell weight than total egg weight. As the birds aged, most parameters increased, while shell thickness decreased. The interaction between housing type and laying period played a crucial role, with egg weight, breadth, and egg shape index increasing as the birds aged. The shell thickness decreased as the laying period progressed across all housing systems. During the 40 to 45 wk period of lay, they exhibited the highest albumen height, haugh unit, and % yolk weight. The study’s findings highlight how the interaction between the housing system and the laying period impacted the internal quality of guinea fowl eggs.

## Introduction

The need for animal protein becomes increasingly important as the world’s population rises. Poultry farming is one of the most efficient and cost-effective methods to satisfy this requirement out of several available options. This is because starting requires very little money, and the birds mature quickly, according to Araújo et al. (2023). Due to the high expense of animal farming, Nigeria’s rural areas see a greater lack of animal protein consumption.

Guinea fowl meat, eggs, and local chicken provide good sources of cheap protein to the rural population (Araújo et al., 2023). This helps to mitigate the effects of poultry product shortages according to Ebegbulem and Asuquo (2018). Guinea fowl (*Numida meleagris*) originated in Africa (John and Malebogo, 2013). *Numida meleagris* and *Numida ptilorhycha* are the two species of guinea fowl native to southern Nigeria’s deciduous rainforest region. Simultaneously, *N. meleagris* is also found in northern Nigeria, and it is expanding to other regions, especially small-scale farming areas (Obike et al., 2011). *Numida meleagris* is one of the main poultry species consumed by many Nigerian families (Ikani and Dafwang, 2004).

Exploration of the existing literature on the guinea fowl welfare and the effects of housing systems on laying potentials as well as the qualities of eggs laid reveals gaps to be filled and suggests that the impact of housing systems on egg quality, particularly in tropical environments, has been understudied, hence the need for further research. Furthermore, the scarcity of information on the influence of housing systems on traits of guinea fowl eggs laid at different laying phases has drawn huge attention because of the potential of guinea fowl as a viable source of cheap animal protein, especially in Nigeria’s rural areas. Guinea fowls are a source of eggs and meat, which consumers value for their taste and nutritional value (Rayan et al., 2022). Guinea fowl eggs are smaller than chicken eggs and have stronger and thicker shells.

The hypothesis that many factors influence some aspect of egg quality from shape, color, and texture of shell to density and color of yolk and mottling of the yolk, seldomly includes the laying phase factor but often excludes the impact of housing systems. Environmental and housing factors such as stocking density, temperature, and humidity play a significant role in shaping egg characteristics. Non-cage systems offer more natural behaviors for hens and potentially improve egg quality. This aligns with existing literature on laying hens and could be explored further in the context of guinea fowl. The relationship between egg and meat quality in guinea fowls could provide a holistic understanding of the overall nutritional value of guinea fowl products. Additionally, changes in housing systems for laying hens over time may have a substantial impact on egg quality, leading to recommendations for sustainable and welfare-conscious housing practices for guinea fowl production. Research on guinea fowl production in tropical environments reveals variations in egg quality attributes at different laying phases. Non-cage systems, such as deep litter, offer more natural behaviors, positively impacting hen well-being. The study also suggests that different laying phases may exhibit variations in egg quality attributes, influenced by the type of housing system used. These findings can help optimize guinea fowl production systems, address protein needs in rural areas, and promote sustainable practices in poultry farming.

Rayan et al. (2022) recently evaluated the quality of guinea fowl eggs. When the comparative study of egg and meat quality of guinea fowl was evaluated, Manyeula et al. (2020) showed that the eggs from lavender guinea fowl had the highest values in terms of weight, shape index, shell thickness (ST), shell percentage, albumen height, and yolk height. For many years, the welfare effects of non-cage vs. cage systems for laying hens have been discussed. The minimal enclosure requirements for laying hens, pullets, and layer breeders are presented, particularly in Europe because they are considered to be more respectful to animal welfare than cage housing systems, which could allow behavioral freedom and promote eco-friendliness (Nielsen, 2023). The prohibition on housing hens in conventional cages has prompted a search for better non-cage housing systems.

Several studies have evaluated deep and deep litter’s effects on run-on poultry production (Oke et al. 2014, 2015, 2016; Abdourhamane & Petek, 2022). However, information is scarce on the impact of the housing system on the egg traits of guinea fowls at different laying phases in tropical environments for an extended period. Although non-cage systems allow for more opportunities for natural behavior than cage systems, environmental monitoring is more important. Therefore, this study aimed to evaluate how different housing systems may impact the external and internal egg quality of guinea fowl hens at different laying phases (periods). It was hypothesized that different housing systems and laying phases would impact the egg quality traits of guinea fowls.

## Materials and Methods

### Experimental Site

The experiment was conducted at the Directorate of University Farms (DUFARMS) Federal University Abeokuta Nigeria. The farms lie within the rainforest vegetation zone of South-Western Nigeria on latitude 7°S 13ʹ49″N, longitude 3° 26ʹ11″E, and have an altitude of 76 m above sea level (Google Earth, 2019). The area is predominantly a hot environment with a humid tropical climate with a mean annual rainfall of about 1,037 mm with most of it falling between April and October a temperature of 34.7 °C and relative humidity of 83%.

### Experimental Birds and Design

This study used 117, 34-wk-old guinea fowl hens. They were randomly assigned to three experimental groups, involving battery cages, where the floor was a wire net supported by metal bars. Each cage was equipped with galvanized wire, deep litter housing systems, and deep litter housing systems with free runs, which had the same dimensions as deep litter houses but with free run and open-air space to allow the birds to have free access to soil and exhibit natural behavior. The experiment was arranged in a 2 × 3 factorial whereby each housing system was replicated thrice with thirteen hens per replicate group and divided into three phases: 36 to 40, 41 to 45, and 46 to 50 wk of age, in line with the laying pattern of guinea fowl.

### Experimental Diets

The feed ingredients were purchased, milled, and processed from a reputable commercial feed mill (Table 1). Feed and water were provided ad libitum. The proximate chemical composition (Table 1) of the diet was done according to the methods of AOAC (1990). The available phosphorus was estimated as 0.3% of the total phosphorus (Shastak and Rodehutscord, 2013), and the metabolizable energy was estimated using the method of Pauzenga (1985).

**Table 1.** Composition (g/kg) of experimental diet for guinea fowl hens

Ingredient	Composition
Maize	440.00
Soybean meal	80.00
Fish meal (72% CP)	20.00
Groundnut cake	75.00
Wheat offal	265.50
Bone meal	40.00
Oyster shell	71.00
Lysine	1.50
Methionine	2.00
^*^Vitamin–mineral premix	2.50
Salt (NaCl)	2.50
Total	1,000.00
Chemical composition	
Metabolizable energy, MJ/kg	10.61
Crude protein, g/kg	16.76
Crude fibre, g/kg	4.27
Ether extract, g/kg	3.87
Calcium, g/kg	3.77
Available phosphorus, g/kg	0.57

^*^1 kg contains: vitamin A: 10,000,000 IU; vitamin D3: 2,500,000 IU; vitamin E:20,000 mg; vitamin K3:. 3,000 mg; vitamin B3:3,000 mg; vitamin B2: 7,000 mg; vitamin B6: 5,000 mg; vitamin B12: 25 mg; biotin:20 mg:. niacin: 15,000 mg; panthotenic acid: 10,000 mg; folic acid:8,500 mg; manganese:80,000 mg; zinc:60,000 mg; iron:40,000 mg; copper:8,000 mg; iodine:. 1,000 mg; selenium (1%): 150 mg; cobalt:250 mg; choline:200,000 mg and antioxidant:. 100,000 mg.

## Data Collection

### Egg External and Internal Quality Traits

A total of 288 eggs were collected and used for the analysis of egg quality traits, whereas six eggs (two eggs per replicate) from each treatment were sampled weekly for the analysis of the egg’s external and internal quality. The collection of eggs commenced when the hens were 36 wk old in June. Eggs were collected for 15 consecutive weeks in the morning between 9:00 and 10:00 a.m. for egg quality trait analysis per treatment.

Quality assessments on external and internal egg contents were performed within 24 h of laying. The eggs used for egg quality assessment were individually numbered and then weighed (Mettler—Toledo® PB3002 electronic balance, USA) to determine their weights to the nearest 0.01 g. The egg breadth and length were measured with a 0.01-mm accuracy Vernier caliper, and these values were used to calculate the egg-shape index, which was calculated as breadth/length × 100, according to Panda (1996). Egg ST, eggshell weight (SW), percentage of SW relative to total egg weight (%SW), and shell surface area (SSA) were all evaluated in egg external quality analyses where SW is the egg SW (g) and the eggSSA was calculated as *S* = (3.155 to 0.0136*L* + 0.0115*B*)*LB*, in which both *L* and *B* were taken in millimeters (Narushin 2005). To avoid contamination, the eggs for internal quality analysis were broken into a small sterile preweighed glass container to obtain the albumen height, albumen weight, albumen percentage, albumen pH, haugh unit, yolk height, yolk weight, and yolk color using the Roche yolk color fan. With a p6085 tripod spherometer with an accuracy of 0.01 mm, the albumen height was measured off the chalazae at a point above midway between the inner and outer circumferences of the thick white.

For individual egg samples, the weight of albumen was calculated as the difference between the egg weight and the combined weight of the yolk and dried eggshell.

% Albumen weight = Albumen weight

Egg weight

The Haugh unit was calculated using the formula based on the egg weight and albumen height of individual egg samples of Haugh (1937), enunciated by Asuquo et al. (1994) in the formula:

HU = log (*H* + 7.5 to 1.7*W*^0.37^)

where HU is Haugh unit, *H* is Albumen height in millimeter, and *W* is the egg weight (g). An egg yolk separator was used to separate the yolk and albumen. Before weighing and measuring the ST, the empty shells were allowed to dry in the open air for 24 h. The empty shells and shell membranes were weighed together.

### Statistical Analysis and Design

The data presented a normal distribution. Data were presented as percentages (egg albumen and yolk) and were arcsine transformed before being analyzed for normalization. Data obtained were arranged and subjected to analysis of variance in a 2 × 3 factorial experimental arrangement (2 factors namely; housing and laying phases, at three levels made up of three housing types and three laying phases respectively). Analyses were done using SPSS 20. Statistically significant mean differences were separated using the Tukey test contained in the statistical package at a 5% level of significance.

## Results

### Effects of Housing System and Period of Lay on External Quality of Guinea Fowl Eggs

#### Main effects of housing type and laying period.

Housing type and laying period significantly (*P* < 0.05) affected all the external egg quality parameters measured (Table 2). The guinea fowls raised on the deep litter housing system recorded the highest egg weight, egg breadth, and SSA values. The SW was similar in guinea fowls raised under the deep litter (5.78 g) and battery cage (5.79 g) housing systems. However, the battery cage housing system yielded the highest ST and percentage of SW relative to total egg weight.

**Table 2.** Effects of housing type and laying period on external quality of guineafowl eggs

		EW, g	EL, mm	EB, mm	ESI	SW, g	ST, mm	%SW	SSA
*Housing type*									
Deep litter		36.80^a^	39.99^c^	33.62^a^	78.40^b^	5.78^a^	0.57^b^	15.75^b^	14.37^a^
Battery cage		35.94^b^	44.57^a^	32.70^c^	77.39^c^	5.79^a^	0.59^a^	16.10^a^	7. 61^c^
Free run		35.13^c^	40.75^b^	32.74^b^	78.76^a^	5.42^b^	0.49^c^	15.43^c^	12.32^b^
*Phase*									
36 to 40 wk		33.52^c^	37.94^c^	26.22^c^	72.47^c^	5.15^c^	0.64^a^	15.44^a^	10.31^c^
40 to 45 wk		36.24^b^	44.32^a^	35.97^b^	80.88^b^	5.78^b^	0.54^b^	15.93^c^	11.44^b^
45 to 50 wk		38.13^a^	43.05^b^	36.86^a^	81.20^a^	6.06^a^	0.46^c^	15.89^b^	12.55^a^
*Interaction*									
Deep litter	36 to 40 wk	34.44^g^	36.21^h^	27.83^g^	74.27^g^	5.37^e^	0.67^a^	15.71^d^	12.87^e^
	40 to 45 wk	37.24^d^	45.02^b^	36.25^d^	80.35^d^	5.84^d^	0.55^c^	15.71^d^	14.62^b^
	45 to 50 wk	38.73^a^	38.74^g^	36.77^b^	80.58^c^	6.13^a^	0.50^d^	15.82^c^	15.62^a^
Battery cage	36 to 40 wk	33.69^h^	43.76^e^	25.75^h^	72.34^h^	5.11^g^	0.67^a^	15.22^g^	9.33^f^
	40 to 45 wk	36.45^e^	44.45^d^	35.71^f^	79.60^f^	6.15^a^	0.59^b^	16.88^a^	5.94^i^
	45 to 50 wk	37.70^c^	45.51^a^	36.64^c^	80.23^e^	6.10^b^	0.50^d^	16.20^b^	7.57^h^
Free run	36 to 40 wk	32.42^i^	33.85^i^	25.09^i^	70.81^i^	4.97^h^	0.59^b^	15.39^f^	8.74^g^
	40 to 45 wk	35.03^f^	43.49^f^	35.96^e^	82.69^b^	5.34^f^	0.49^d^	15.21^g^	13.76^d^
	45 to 50 wk	37.95^b^	44.92^c^	37.17^a^	82.78^a^	5.94^c^	0.39^e^	15.65^e^	14.45^c^
	SEM	0.400	2.814	0.955	0.832	0.086	0.017	0.097	0.657
*P-values*	Housing	0.000	0.000	0.000	0.000	0.000	0.000	0.000	0.000
	Phase	0.000	0.000	0.000	0.000	0.000	0.000	0.000	0.000
	Interaction	0.000	0.000	0.000	0.000	0.000	0.000	0.000	0.000

^a–c; a–i^Means with different superscripts within the same column are significantly different (*P* < 0.05).

EW, egg weight; EL, egg length; EB, egg breadth; ESI, eggshell index; SW, shell weight; ST, shell thickness; %SW, % shell weight; SSA, shell surface area.

The values of egg weight, egg breadth, eggshell index, SW, and SSA increased (*P* < 0.05), while the ST was observed to be thinner (*P* < 0.05) as the birds aged. The other parameters (egg length and %SW) followed no particular trend with the guinea fowls’ period of lay.

#### Interaction between housing type and period of lay.

All the parameters measured were significantly (*P* < 0.05) affected by the interaction between housing systems and the period of lay on the external parameters of guinea fowl eggs, as shown in Table 2. For all housing types, the egg weight, egg breadth, and egg shape index increased as the guinea fowls aged, with the highest values for egg weight (38.73 g), egg breadth (37.17 mm), and egg shape index (82.78) recorded for deep litter (45 to 50 wk), free run (45 to 50 wk), and free run (45 to 50 wk), respectively. The ST decreased as the laying period progressed for all housing types. The intensive rearing (Deep litter and battery cage) systems elicited the thickest shell in the guinea fowls, albeit at 36 to 40 wk of lay. The SSA was also observed to increase as the period of the lay of the guinea fowls increased.

### Effects of Housing System and Period of Lay on Internal Quality of Guinea Fowl Eggs

#### Main effects of housing type and laying period.

All the internal egg quality parameters of guinea fowl considered in this study were significantly (*P* < 0.05) affected by the housing type and laying periods (Table 3). Albumen weight, yolk weight, and % yolk weight of the guinea fowls’ eggs were highest with those raised under the deep litter housing system. The birds raised on the free-run housing system recorded the highest haugh unit, best yolk color, and albumen pH, although the albumen pH of the eggs from the three different housing systems tended to be alkaline (but within the 8.0 range). The battery cage housing system elicited the highest albumen height and % albumen in the guinea fowls. The albumen weight, yolk weight, and albumen pH of the guinea fowls were observed to increase as the birds aged while the yolk height value decreased. The albumen height (6.73 mm), haugh unit (89.33), and % yolk weight (28.90%) were highest during the 40 to 45 wk period of lay.

**Table 3.** Effects of housing type and laying period on internal quality of guineafowl eggs

		AH, mm	AW, g	% ALB	HU	YES	YW, g	%YW	YH, mm	ApH
*Housing type*										
Deep litter		6.49^c^	18.26 ^a^	50.69^b^	87.71^b^	1.57^b^	16.44^a^	28.88^a^	13.74^b^	8.62^b^
Battery cage		6.67^a^	18.01^b^	50.92^a^	89.16^c^	1.49^c^	9.10^c^	28.29^b^	13.77^a^	8.55^c^
Free run		6.61^b^	17.61^c^	45.85^c^	87.53^a^	3.10 ^a^	10.06^b^	28.68^c^	13.75^c^	8.64^a^
*Phase*										
36 to 40 wk		6.36^c^	16.80^c^	50.46^b^	88.15^b^	1.46^b^	9.44^c^	26.64^b^	13.84^a^	8.23^c^
40 to 45 wk		6.73^a^	17.82^b^	50.03^c^	89.33^a^	1.63^c^	10.35^b^	28.90^a^	13.75^b^	8.70^b^
45 to 50 wk		6.68^b^	19. 25^a^	50.97^a^	86.91^c^	3.08^a^	16.70^a^	28.30^c^	13.68^c^	8.89^a^
*Interaction*										
Deep litter	36 to 40 wk	6.41^e^	17.18^g^	50.98^d^	88.10^e^	0.99^g^	9.72^g^	28.98^c^	14.06^c^	8.25^g^
	40 to 45 wk	6.51^d^	18.29^d^	50.57^e^	87.78^f^	1.99^c^	10.46^d^	28.46^e^	13.67^e^	8.80^c^
	45 to 50 wk	6.54^c^	19.30^b^	50.53^f^	87.24^g^	1.74^d^	29.15^a^	29.19^b^	13.50^f^	8.81^c^
Battery cage	36 to 40 wk	6.51^d^	17.07^h^	51.42^a^	89.22^c^	1.32^e^	9.44^h^	28.51^d^	13.52^f^	7.86^h^
	40 to 45 wk	6.76^b^	17.84^e^	50.16^g^	89.45^b^	1.16^f^	9.99^f^	28.05^h^	14.44^a^	8.46^f^
	45 to 50 wk	6.75^b^	19.11^c^	51.17^c^	88.80^d^	1.99^c^	10.54^c^	28.30^g^	13.36^g^	9.34^a^
Free run	36 to 40 wk	6.16^f^	16.15^i^	48.99^i^	87.14^h^	2.07^b^	9.17^i^	28.42^f^	13.93^d^	8.57^d^
	40 to 45 wk	6.92^a^	17.34^f^	49.37^h^	90.75^a^	1.73^d^	10.59^b^	30.20^a^	13.14^h^	8.84^b^
	45 to 50 wk	6.75^b^	19.34^a^	51.20^b^	84.70^i^	5.50^a^	10.42^e^	27.42^i^	14.19^b^	8.52^e^
	SEM	0.042	0.209	0.156	0.320	0.249	1.182	1.145	0.006	0.077
*P-values*	Housing	0.000	0.000	0.000	0.000	0.000	0.000	0.000	0.000	0.000
	Phase	0.000	0.000	0.000	0.000	0.000	0.000	0.000	0.000	0.000
	Interaction	0.000	0.000	0.000	0.000	0.000	0.000	0.000	0.000	0.000

^a–c; a–i^Means with different superscripts within the same column are significantly different (*P* < 0.05).

EW, egg weight; AH, albumen height; AW, Albumen weight; %ALB, %Albumen; HU, Haugh unit; YC, yolk color; YW, yolk weight; %YW, %yolk weight; YH, yolk height; ApH, albumen Ph.

#### Interaction between housing type and period of lay.

The interaction between housing systems and the period of lay significantly (*P* < 0.05) affected all the external quality parameters of guinea fowl eggs measured in the study (Table 3). The birds raised on free-run housing systems during the 40- to 45-wk laying period recorded the highest albumen height (6.92 mm), haugh unit (90.75), and % yolk weight (30.30%). The albumen weight increased consistently as the period of lay increased for all housing types. Although the haugh unit and yolk height values were inconsistent across the treatments, a reduction in values was observed as they aged for the deep litter housing system, while the other housing systems also had a slightly similar trend.

## Discussion

The effect of the housing system and period of lay on both the external and internal quality parameters of guinea fowl eggs revealed that both factors had a significant effect on the egg quality, with different housing systems and laying periods influencing different aspects of the eggs’ external and internal characteristics. Guinea fowls raised on the deep litter housing system exhibited the highest egg weight, egg breadth, and SSA values. Dikeir Kogoor et al. (2021) suggested that the deep litter system may provide a favorable environment that promotes better egg development. In the present study, the battery cage housing system yielded the thickest eggshells and the highest percentage of SW relative to the total egg weight. This is in tandem with the study by Ketta and Tumova (2016), who indicated that the battery cage system influenced the mineralization of the eggshell, resulting in thicker, and stronger shells.

The laying period has also had a significant influence on external egg quality parameters. The egg weight, egg breadth, eggshell index, SW, and SSA increased while the thickness of the shell decreased as the guinea fowls aged. This pattern is consistent with the observation of Marinko et al. (2018), who reported the typical egg development patterns of grey guineas in terms of certain egg qualities. Qiu et al. (2020) proposed that a decrease in egg ST may be caused by mineral depletion or changes in the hen’s metabolism as it continues to lay eggs. Joyner et al. (1987) opined that older birds typically lay heavier, bigger eggs with thinner shells.

The interaction between the housing system and the laying period further impacted external egg quality parameters. Egg weight, egg breadth, and egg shape index increased with guinea fowl age for all housing types. The deep litter housing system produced the highest values for egg weight, egg breadth, and egg shape index during the 45- to 50-wk laying period in this study. This finding shows that deep litter housing and specific laying period create conditions that favor the production of bigger, more rounded eggs, which is tandem with the study by Shoyombo et al. (2021), who stated that the deep litter housing systems, which give guinea fowls a more natural habitat, typically produce eggs with greater average egg weights, lengths, and shapes.

Other internal egg quality measures were also significantly influenced by the housing type; the deep litter housing system gave rise to guinea fowls that laid eggs with the highest albumen weight, yolk weight, and percentage of yolk weight in the study by Dahloum et al. (2018), who stated that deep litter system might provide more nutrients and stimulate higher albumen and yolk development, which is shown by this result, which improves the potential for it. On the other hand, the free-run housing system produced eggs with the best albumen pH, highest haugh unit, and best yolk color. Nemati et al. (2020) opined that these parameters are important indicators of egg freshness and quality.

Compared to external quality, internal egg characteristics were significantly influenced by the laying period. As the birds aged, the yolk height declined while the albumen weight, yolk weight, and albumen pH increased. These patterns reflect the typical variations noticed during egg maturation in the study conducted by Marzec et al. (2019), who stated that the egg absorbs more nutrients, and albumen, as yolk weight increases but yolk height declines as a result of water loss.

The interaction between housing type and laying period also influenced internal egg quality parameters. For instance, during the 40- to 45-wk laying phase, guinea fowls raised in free-run housing had the maximum albumen height, Haugh unit, and percentage of yolk weight. This finding implies that the internal egg quality may be optimized by a particular housing system and laying period. On the contrary, the study by Berrama et al. (2021) stated that the laying period did not affect the internal characteristics of Japanese quail eggs.

## Conclusion

Premised on the results obtained, it was concluded that housing type and laying period significantly influenced egg quality parameters, with deep litter housing exhibiting higher egg weight, egg breadth, and SSA. Battery cage housing had thicker shells and a higher percentage of SW than total egg weight. As the birds aged, most parameters increased, while ST decreased. The interaction between housing type and laying period played a crucial role, with egg weight, breadth, and egg shape index increasing as the birds aged. The ST decreased as the laying period progressed across all housing systems. The interaction between housing type and laying period further underscored the complexity of factors influencing egg quality, with certain parameters exhibiting notable changes as the laying period progressed.
